# Novel Methylation Patterns Predict Outcome in Uveal Melanoma

**DOI:** 10.3390/life10100248

**Published:** 2020-10-20

**Authors:** Sarah Tadhg Ferrier, Julia Valdemarin Burnier

**Affiliations:** 1 Cancer Research Program, Research Institute of the McGill University Health Centre, Montreal, QC H4A 3J1, Canada; sarah.ferrier@mail.mcgill.ca; 2 Experimental Pathology Unit, Department of Pathology, McGill University, Montreal, QC H3A 0G4, Canada; 3 Department of Oncology, McGill University, Montreal, QC H3A 0G4, Canada

**Keywords:** epigenetics, DNA methylation, uveal melanoma, BAP1, prognostic markers, metastasis

## Abstract

Uveal melanoma (UM) is the most common intraocular tumor in adults. Despite effective local treatments, 50% of patients develop metastasis. Better ways to determine prognosis are needed as well as new therapeutic targets. Epigenetic changes are important events driving cancer progression; however, few studies exist on methylation changes in UM. Our aim was to identify methylation events associated with UM prognosis. Matched clinical, genetic, and methylation data for 80 UM cases were obtained from The Cancer Genome Atlas (TCGA). Top differentially methylated loci were sorted through hierarchical clustering based on methylation patterns, and these patterns were compared to tumor characteristics, genomic aberrations, and patient outcome. Hierarchical clustering revealed two distinct groups. These classifications effectively separated high and low-risk cases, with significant differences between groups in patient survival (*p* < 0.0001) and correlation with known prognostic factors. Major differences in methylation of specific genes, notably *NFIA*, *HDAC4*, and *IL12RB2*, were also seen. The methylation patterns identified in this study indicate potential novel prognostic indicators of UM and highlight the power of methylation changes in predicting outcome. The methylation events enriched in the high-risk group suggest that epigenetic modulating drugs may be useful in reducing metastatic potential, and that specific differentially methylated loci could act as biomarkers of therapeutic response.

## 1. Introduction

Uveal melanoma (UM) is the most common primary intraocular tumor in adults and the most common non-skin form of melanoma, with a reported incidence of 5.1 cases per million in the US [1]. Unlike cutaneous melanoma (CM), UM arises from melanocytes located in the uveal tract, most commonly in the choroid, and displays different genomic mutations and molecular profile than the more common CM [2]. Despite effective methods for treating the primary tumor–either through local radiotherapy or less commonly enucleation [3]—there is currently no effective treatment for metastatic disease, which occurs in approximately 50% of patients regardless of primary ocular treatment [4]. Unfortunately, metastatic UM is associated with high mortality within 6–12 months.

Prognostic factors of UM include features such as cell type, with a poorer prognosis in patients with epithelioid cell tumors and better prognosis for spindle cell tumors as well as tumor size. Additionally, tumors with increased mitotic activity, closed vascular loops in the tumor, increased tumor-infiltrating lymphocytes, and extrascleral extension all show poorer outcomes [5]. Genetically, UM is characterized by a set of chromosomal aberrations and somatic mutations. Most notably, monosomy in chromosome 3 is an important prognostic marker related to metastasis [5,6]. Copy number variations in chromosome 6 and 8 are also observed, with gain of chromosome 8q being associated with poor prognosis [7]. More than 80% of UMs harbour mutually exclusive mutations in *GNAQ* or *GNA11*, which lead to constitutive activation of signaling pathways such as the RAS-ERK and PI3K/AKT/mTOR pathways. *GNAQ* and *GNA11* mutations are initiating mutations in UM, and despite their presence in almost all of these tumors, the presence of these mutations is not generally related to the development of metastasis or to prognosis [8]. There are three mutations that have been shown to be associated with prognosis in UM; *EIF1AX*, *SF3B1*, and *BAP1* mutations can be used to classify UMs into low, intermediate, and high-risk, respectively. *EIF1AX* mutations are generally an indicator of good prognosis and are associated with low risk of metastasis. *SF3B1* mutations have been associated with late metastases, while *BAP1* mutations are associated with the development of early metastases [9]. While there is research on the genetic changes that underlie metastasis in UM, little is known about the mechanisms by which systemic progression occurs, and more work is needed to uncover the mechanisms through which the genetic aberrations seen in UM lead to metastasis.

DNA methylation is an important and well-studied epigenetic modification in mammals that is normally responsible for the regulation of gene expression, especially in developing cells. This process is regulated by DNA methyltransferases, which are responsible for maintaining DNA methylation on the genome. DNA methylation is associated with alterations in chromatin structure, and methylation occurring specifically on CpG islands is highly associated with the silencing of gene expression [10]. DNA methylation is recognized as an important event in cancer, where a pattern of global hypomethylation leading to genomic instability is often seen [11]. Along with this, many tumor types also show a specific pattern of hypermethylation at CpG islands which can be important in tumor progression, for example leading to the silencing of tumor suppressor genes [12]. While the mechanisms underlying tumor methylation are not fully understood, changes in methylation are maintained throughout cell replication and play an important role in the progression of multiple tumor types [13,14]. Because epigenetic processes such as DNA methylation are mitotically heritable, they can play similar roles as genetic alterations in the development of cancer, making them an important target in both prognostication and drug development. This is especially important as epigenetic events can confer growth advantages to cells by disrupting gene expression similarly to genetic events, but can exert its effects much more rapidly than mutations [15].

In UM, several known changes in promoter methylation have been studied, such as on the *RASSF1A* gene [16], and a global pattern of methylation has been associated with molecular subtype and overall prognosis [16,17]. Additionally, a recent study has shown that BAP1 knockdown in UM cells is associated with methylomic reprogramming in these cells, pointing to a link between the genetic mutations seen in UM and large scale changes in methylation pattern [18]. Despite these observations, few studies have investigated whether the changes in methylation pattern may contribute to the metastatic phenotype. As such, further investigation into the specific changes in methylation seen in these tumors are needed to more completely uncover the events that dictate outcome in UM and to uncover new therapeutic targets. Given the promise that epigenetic-targeting agents have shown in many tumor types, either through targeting specific modifications directly or through targeting epigenetic regulators, the reversal of epigenetic alterations may be a promising avenue for preventing metastasis in UM [19,20]. Furthermore, monitoring the specific changes in UM methylation that are associated with a high risk of metastasis would indicate tumor response to different therapeutic agents. This is particularly important given the high rate of metastasis in UM and its poor prognosis.

In this study, we sought to investigate in detail potential epigenetic biomarkers in UM that could be related to prognosis. Using the Cancer Genome Atlas (TCGA) data, we were able to differentiate two groups with prognostically significant patterns of methylation, and to highlight some potential targets for high risk of metastasis in UM. We demonstrate the importance of specific methylation changes in UM on tumor progression by looking at both the overall promoter methylation pattern as well as at specific loci which are highly differentially methylated depending on the risk level of the patient. The data represents an important step in determining promising targets for better prognostication and treatment in this deadly ocular malignancy.

## 2. Methods

### 2.1. Dataset

Raw methylation intensity values from the Illumina 450k methylation array were obtained directly from the TCGA legacy archive for 80 UM cases. Along with this, the *TCGA biolinks* package was used to extract extended clinical data, RNAseq, and copy number variation (CNV) files. The Illumina 450k array annotation was obtained with the information from hg19 human genome assembly to map the loci on the array to their genomic location.

### 2.2. Removal of Poor-Quality Probes

The *Minfi* program was used to read the raw IDAT files and to calculate the detection *p* value for every genomic position in each of the 80 samples. Positions with *p* values > 0.01 were discarded from further analysis to remove any probes where both the methylated and unmethylated channels reported background signal level, as determined by the negative control positions in the array. The mean detection *p* values across all samples were also calculated in order to ensure that there were no poor quality samples [21]. Probes that failed in one or more samples were removed from further analysis (total of 6480 removed), as well as probes on sex chromosomes, in order to remove some of the variation in methylation pattern caused by sex differences (11,004 removed). A total of 467,668 probes were kept for further analysis.

### 2.3. Normalization of Samples

The normalization was performed through both Quantile and SWAN normalization for further comparison [22,23]. These values were compared to the raw data, obtained using the *preprocessRaw* function, which brings together the methylated and unmethylated channels into beta values without further normalization. Quality Control reports were produced using Minfi and the different methods of normalization were visually compared using these reports.

### 2.4. Hierarchical Clustering of the Top Differentially Methylated Probes (DMPs)

The standard deviation across samples for each probe was calculated, and they were subsequently sorted by degree of differential methylation, as defined by the points which had the highest standard deviation across samples. The top 10,000 of these DMPs were selected for further analysis. These probes were inputted into Minfi in order to perform hierarchical clustering.

The results of this hierarchical clustering were compared to selected clinical and genetic data in order to determine whether the groups were clustering based on prognostically relevant data. The two groups were compared based on the sex and age of the individuals, survival and the development of metastatic UM, *GNAQ*, *GNA11*, and *BAP1* mutations, and CNVs.

### 2.5. Analysis of DMPs between the Two Groups

DMPs were also analysed using limma, which compared these DMPs across the previously determined groups from the hierarchical clustering, with the probes aligned to the hg38 human genome assembly. The highly differentially methylated probes, considered as those with a log fold change of ±1.5 or more in between the groups, were submitted into the DAVID Functional Annotation Clustering Tool.

Custom JavaScript code was used to separate CpGs for genes of interest in order to gather the quantile normalized beta values for all DMPs on the CpGs associated with these genes in all cases.

## 3. Results

### 3.1. Similar Levels of Overall Methylation Are Seen in 80 Cases of UM through Analysis of TCGA Data

**Figure 1:**
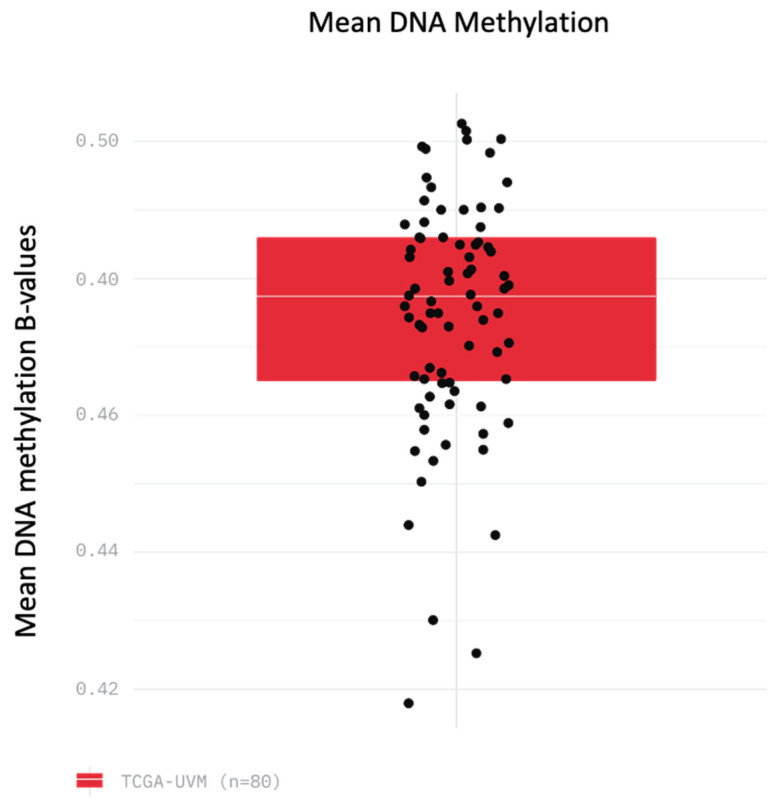
Total mean DNA methylation values for each patient sample (n = 80) as calculated by mean methylation beta values (ratio of intensities between methylated and unmethylated alleles) for all probes in the Illumina 450k methylation array.

A total of 80 UM cases from the TCGA were analysed for methylation profiling. Patient characteristics can be seen in Table 1. After removal of poor-quality probes and normalization of data, the overall level of methylation at the remaining sites was analyzed. Overall, the cases showed very similar levels of overall methylation, with an average ratio of intensities between methylated and unmethylated alleles (beta value) of 0.48 (range = 0.42–0.5) (Figure 1). This suggests that any differences between these samples are not due to higher or lower overall methylation at all probes, but instead are caused by hypermethylation and hypomethylation at specific CpG islands. Of the probes studied, 1708 probes were found to have a log fold change value of at least 2.0 (range = 2.00 to 4.73), while 785 probes were found to have log fold change values of −2.0 or less (range = −2.00 to 4.17).

### 3.2. Unsupervised Clustering Analysis Reveals Two Main Methylation Patterns in This Cohort

**Figure 2:**
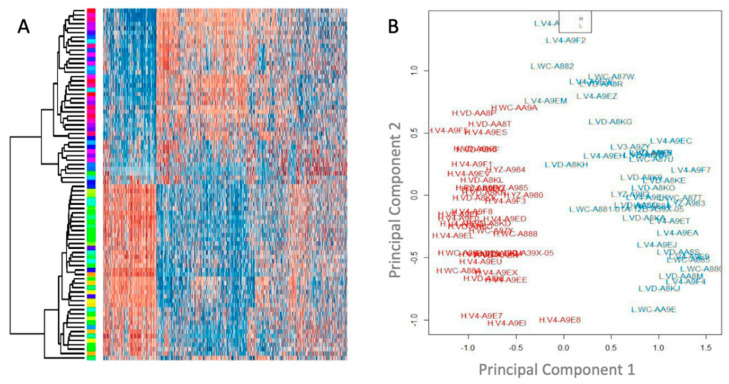
Unsupervised clustering analysis of 80 Uveal Melanoma (UM) cases from TCGA. (**A**) Heatmap showing the top 10,000 loci for all the patients using quantile normalized beta values, with dark red being fully hypermethylated and dark blue being fully hypomethylated for each locus. (**B**) Principal component analysis for cases, labeled by case number and risk group (as determined by hierarchical clustering).

As overall degree of methylation was similar across cases (Figure 1), we aimed to determine whether patterns of methylation at specific sites would reveal important differences in the patient cohort. Unsupervised clustering of these 80 cases of UM based on the pattern of differential methylation revealed two major groups as shown in a heat map (Figure 2A) and principal component analysis (Figure 2B).

### 3.3. Methylation Patterns Are Significantly Associated with Outcome and Effectively Stratify Patients into High and Low Risk Groups

**Figure 3:**
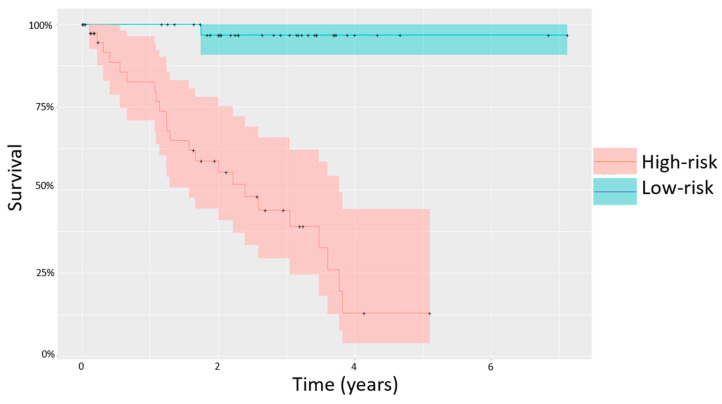
Comparison of the two groups of patients determined by hierarchical clustering. Kaplan–Meier survival function for the patients based on methylation risk groupings.

Upon further investigation into these groups, no statistically significant differences were seen in clinical features that are generally prognostically insignificant, with similar M:F sex ratios (*p* = 0.822) and mean age at diagnosis (59 vs. 65 years, *p* = 0.423). In contrast, the two groups identified by unsupervised clustering analysis differed significantly in terms of outcome, and we therefore termed them “high-risk” and “low-risk” based on the clinical outcomes of the patients in each group (Table 2). Importantly, overall survival varied very significantly between the two groups as shown by a Kaplan-Meier curve (Figure 3). While 23 (58%) of the 40 high risk patients developed metastasis, only 4 (10%) of the low risk patients did (*p* < 0.00001, Table 2). Of these 4 patients, only 1 died, while 22 of 23 metastatic patients in the high-risk group died (*p* < 0.00001; average survival of 1.79 years before death from metastatic UM, Table 2).

### 3.4. The High and Low-Risk Groups Differed in Clinical and Histopathological Features of the Ocular Tumors

Like in many malignancies, clinical and histopathological features correlate with patient outcome. In UM, increasing ocular tumor size has been shown to be associated with decreased survival [5]. Moreover, UM can be classified according to cell type: epithelioid, spindle, or mixed cell tumors. While spindle cell tumors have better prognosis, tumors composed of epithelioid cells are associated with worse prognosis [5]. As such, metastatic UM tumors tend to be predominately composed of epithelioid- or mixed-cell populations. Here, the two groups identified in the methylation analysis were compared on the basis of tumor features, including size and cell type. In terms of cell type, the clinical information from TCGA was classified by approximate percentages for each cell type as well as by the number of epithelioid or spindle cell predominant tumors in each group. While both groups contained epithelioid and spindle tumors, the high-risk group showed a higher proportion of epithelioid cell type tumors (20 vs. 3 epithelioid or epithelioid-predominant tumors). In terms of tumor size, tumors of the low-risk group were smaller in both thickness (average thickness = 9.99 mm, range = 5–15.5 vs. 10.85 mm, range = 4–16) and average basal diameter (average diameter = 16.15 mm, range = 7.8–23.6 vs. 17.72 mm, range = 10.6–25), with only diameter showing significance between groups (*p* < 0.05, Table 3). The number of tumors with closed connective loops were also significantly different between groups (*p* < 0.0005, Table 3).

### 3.5. Methylation Stratification Highly Correlated with Genomic Factors Associated with Metastasis

**Figure 4:**
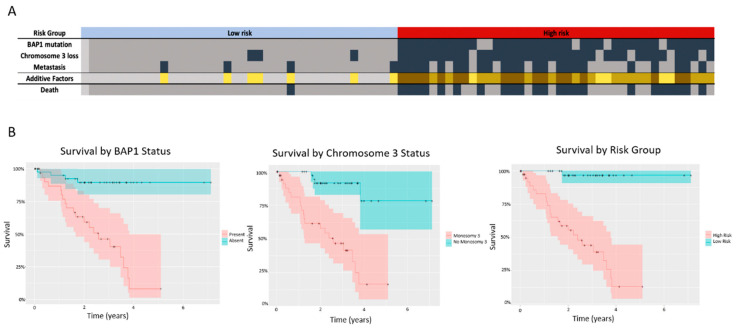
(**A**) Prognostic factors (BAP1 mutation, chromosome 3 loss, and development of metastasis) separated by risk grouping. Total number of these prognostically significant factors present for each patient represented in yellow scale. Death (due to confirmed metastatic disease or deaths with no specified cause) is also shown. Dark grey represents the occurrence of the event. (**B**) Comparison of survival for major prognostic factors BAP1 (*p* = 2.47 × 10^−6^), chromosome 3 loss (*p* = 2.98 × 10^−6^), and methylation risk groupings (*p* = 5.35 × 10^−8^).

Given the relatively short follow up (average of 2.35 years for the patients who did not succumb to metastasis), it is not possible to determine which patients would develop metastasis. Because of this, we compared our methylation groups to known markers of poor prognosis, such as mutations and chromosomal aberrations, to determine the likelihood of metastasis (Figure 4A).

The occurrence of *GNAQ* and *GNA11* mutations, which are initiating events in UM and not generally believed to be prognostically significant, differed between the two groups, with more *GNAQ* mutations in the low risk group (25 vs. 15, *p* < 0.05) and slightly more *GNA11* mutations in the high risk group (22 vs. 14, *p* = 0.07, Table 4). *BAP1* mutations are the most prognostically significant genomic alteration in UM, and are associated with high risk of metastasis [24]. The presence of a *BAP1* mutation was significantly associated with survival in this cohort (*p* = 0.00022, Figure 4B). The two groups differed by the number of cases with presence/absence of a *BAP1* mutation (*p* < 0.00001), with all confirmed *BAP1* mutations (24/80) found in the high-risk group (Figure 4A).

In addition to *BAP1* mutations, chromosomal changes are significantly associated with risk of metastasis and survival in UM [7]. Monosomy 3 is associated with high risk of metastasis and is the strongest cytogenetic indicator to predict UM metastasis [6,7]. Indeed, loss of chromosome 3 was significantly associated with survival (Figure 4B). Loss of chromosome 3 was significantly more common in the high-risk group than in the low risk group (35 cases vs. 3 cases, *p* < 0.00001) (Table 4, Figure 4A).

Moreover, amplification of chromosome 8q, which is found in 40% of UMs, is also associated with poor prognosis [6]. Cases with gains in chromosome 8q in this cohort were associated with high-risk grouping (*p* < 0.00001), while gain of chromosome 6p were associated with the low-risk grouping (*p* < 0.00001, Table 4). Another frequent alteration is chromosome 1p loss (found in 25% of UMs), which occurs frequently with monosomy 3. Loss of chromosome 1p is not associated with decreased disease-free survival except in instances where this loss is combined with a loss of chromosome 3 [7]. Loss of chromosome 1 was similar between risk groupings (Table 4), although it was more commonly seen alongside a loss of chromosome 3 in the high-risk group (10 cases of concurrent loss of chromosomes 1 and 3 in the high-risk group vs. 1 case in the low-risk group).

Importantly, all cases in the high-risk group had at least one important marker of poor outcome (either chromosome 3 loss, confirmed *BAP1* mutation, and/or development of metastasis) (Figure 4A) [7,24]. Additionally, the methylation groupings were more accurate in predicting death from metastasis in this cohort than other known prognostic indicators of UM, including chromosome 3 loss, *BAP1* mutations, and clinical features (Figure 4B).

### 3.6. Gene Ontology (GO) Analysis Reveals Enrichment for Genes Involved in Signal Transduction Pathways in the High-Risk Group

**Figure 5:**
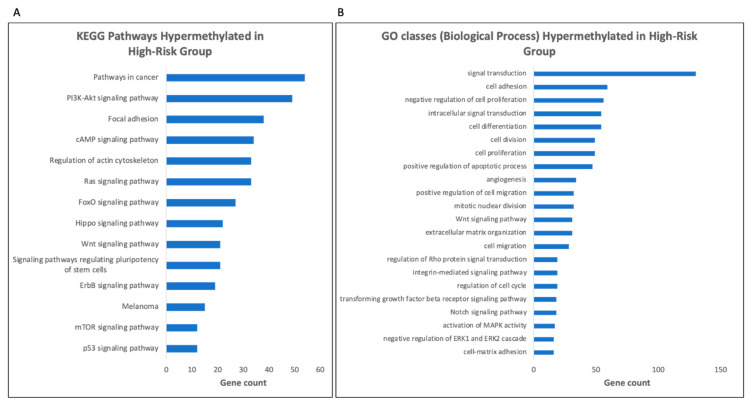
Selected list from the most highly affected Gene Ontology (GO) pathways between the two groups based on differentially methylated genes (log fold change of 1.5× or more between the high and low risk groups) for (**A**) KEGG pathways and (**B**) GO biological process classes. Pathways sorted by number of genes in each class, analysis done in DAVID using EASE score of 0.05.

We conducted a GO analysis to determine gene classes that were commonly differentially methylated between the two risk groups. Our analysis revealed that the most significant DMPs were especially enriched for genes involved in signal transduction, including genes associated with pathways in cancer (KEGG pathways) (Figure 5A, Table S1) and in tumor suppressor genes (Figure 5A, Table S2). In the high-risk group, DAVID GO revealed that many of the genes with the highest log fold change in methylation levels between the two groups were involved in signal transduction such as for mTOR signaling, PI3K-Akt signaling, and RAS signaling (Figure 5B, Tables S3–S5). Along with this, the analysis showed hypermethylation of a number of genes involved in the negative regulation of ERK1/2 in the high-risk group, as seen by analysis of DAVID biological process GO (Table S6). This category includes the hypermethylation of 10 probes associated with in the *PTEN* gene in the high-risk group (Table S7). 

### 3.7. Hypermethylation of Tumor Suppressor Genes and Transcriptional and Epigenetic Regulators Are Seen in the High-Risk Group

**Figure 6:**
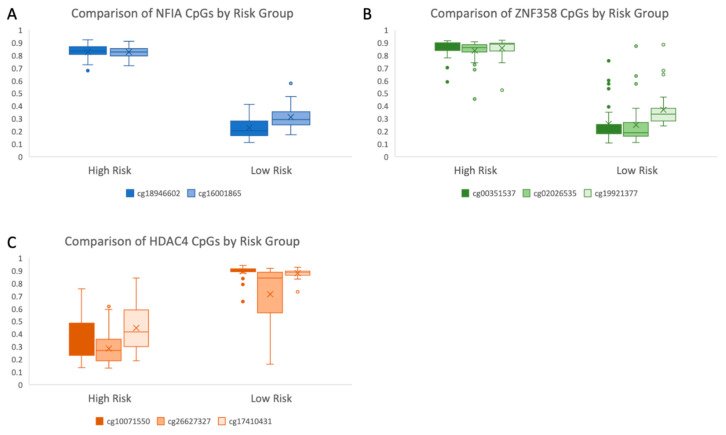
Box and whisker plots of average Beta values for all cases across selected highly differentially methylated probes, separated by high and low risk group, for (**A**) the *NFIA* gene (designated in blue and orange), (**B**) the *ZNF358* gene (designated in blue, orange, and grey) (**C**) the *HDAC4* gene (designated in blue, orange, and grey). Full list of differentially methylated probes for each of these genes available in Supplementary Tables S9, S11 and S13.

Among the many DMPs, several tumor suppressor genes and transcriptional regulators appear to be methylated in the high-risk group. Additionally, genes coding for one of the IL12 receptor subunits, IL12RB2, were found to be hypermethylated in the high-risk group (Table S8). Of interest, a high degree of differential methylation between the two groups was seen at nine probes (with a log fold change of more than ±1.5) thought to be involved in coding of *NFIA* (Figure 6A, Table S9). *RASSF1*, a gene known to be inactivated through methylation in UM, was also found to be highly differentially methylated at multiple probes between the high- and low-risk groups (Table S10). Similarly, hypermethylation in the high-risk group in multiple *ZNF* genes, especially in ZNF358 (three probes with a log FC > 1.5, range: 3.49–4.37, Figure 6B) and ZNF532 (nine probes with a log FC > 1.5, range: 1.63–4.04) was seen (Tables S11 and S12).

### 3.8. Changes in Methylation Are Found in Genes with a Role in Epigenetic Modifications

HDAC inhibitors are currently being investigated in UM for their potential to reverse the phenotypic effects of loss of BAP1 expression [25]. Interestingly, 49 probes associated with HDAC were found to be hypomethylated in the high-risk group, including probes with a log fold change of up to 4.08 between the two risk groups (sample of differentially methylated CpGs, Figure 6C, Table S13).

**Figure 7:**
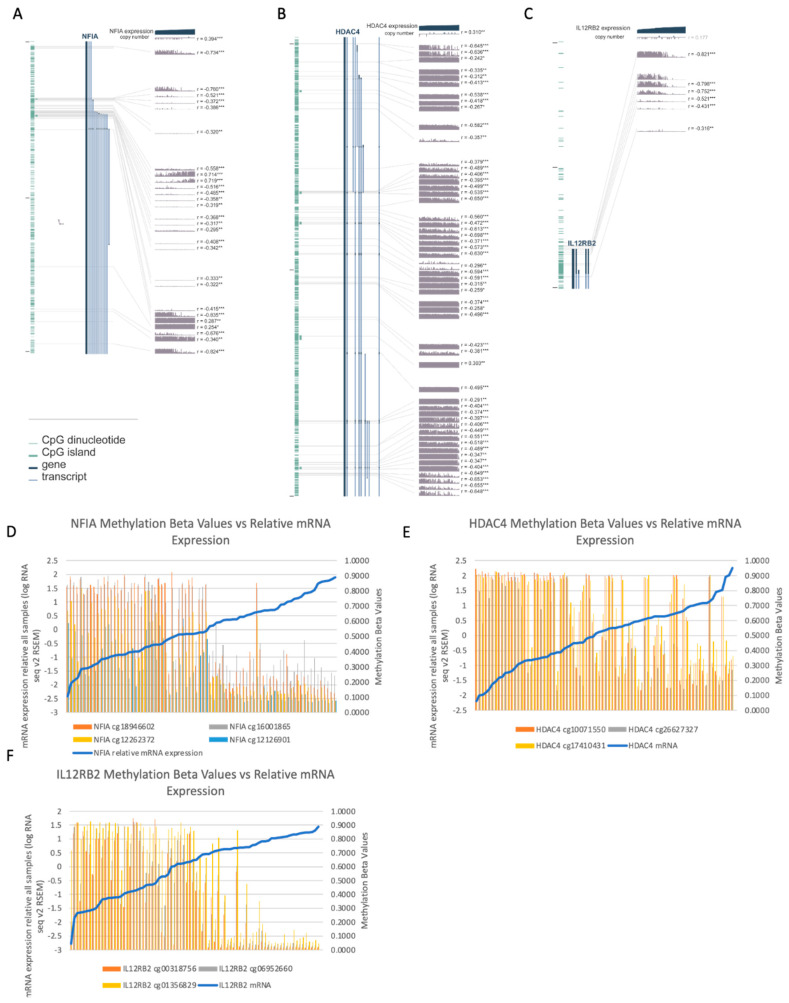
Degree of methylation at selected loci across all cases (n = 80), sorted in order of increasing gene expression (lowest to highest gene expression) for (**A**) the *NFIA* gene (**B**) the *HDAC4* gene and (**C**) the *IL12RB2* gene. For each probe, * *p* < 0.05, ** *p* < 0.01, *** *p* < 0.001. Selected methylation beta values vs. mRNA expression z-scores relative to all samples (mRNA expression data obtained through the cBioPortal for Cancer Genomics as log RNA Seq V2 RSEM) for (**D**) the *NFIA* gene (**E**) the *HDAC4* gene, and (**F**) the *IL12RB2* gene.

Moreover, we assessed the RNA sequencing data to determine if methylation at these genes may be associated with differences in gene expression, which showed that for certain CpG sites for these genes, hypermethylation was associated with reduced expression (Figure 7).

## 4. Discussion

In this study, we conducted an analysis to investigate potential epigenetic biomarkers in UM that could be related to prognosis. Unsupervised clustering analysis of 80 UM cases showed separation of patients into two groups based on methylation changes. These groups were very significantly associated with outcome, highlighting the importance of methylation profiling in this tumor type. The data suggest that differential methylation may act as a predictor of prognosis in UM (Figure 3).

A loss of function mutation in *BAP1* is a very strong predictor of metastasis, and was found in 24 cases of this cohort, all of which were placed into the high-risk group. All cases in the high-risk group had one or more important markers of poor outcome (either confirmed metastatic UM, chromosome 3 loss, and/or confirmed *BAP1* mutation), suggesting that these cases all show high metastatic potential as compared to the cases designated into the low-risk group (Figure 4).

Additionally, the methylation groupings were more accurate in predicting the development of metastasis in this cohort than other significant markers of UM progression, including chromosome 3 loss, *BAP1* mutations, and tumor features used to predict prognosis (Figure 4B). This is clinically significant because of the limitations of known prognostic markers in UM. *BAP1* mutations are difficult to detect as they can occur on multiple locations in the gene, therefore requiring whole-gene sequencing. While immunohistochemistry has been used for BAP1 protein expression profiling, further work remains to be done in this field to confirm the usefulness of BAP1 IHC for prognostication in UM [26]. Additionally, while there are clinical features such as epithelioid cell type, presence of lymphocytic infiltrate, increased mitotic activity, and tumor size that are useful in prognostication, these features can show intra-tumor heterogeneity and depend on the biopsy specimen that is analyzed [27]. As such, methylation patterns may provide a powerful alternative or complementary biomarker of prognosis, requiring small amounts of input DNA. Furthermore, techniques such as liquid biopsy are gaining more interest in detection of tumor biomarkers, and even with fragmented and low abundance DNA, can be used to detect methylation patterns [28]. Our group is currently developing such an approach in UM. In recent years, gene expression profiling (GEP)-based testing has become more important for prognostication of UM, based on the development of a 15-gene panel validated by Onken et al. This test has been shown to be a strong indicator of prognosis, dividing 446/459 of studied patients into class 1 and class 2 GEP, with 1.1% of class 1 patients and 25.9% of class 2 patients developing metastases after a median follow-up of 17.4 months [29].

Three of the four patients in the low-risk group who developed metastasis were alive as of the most recent updates in the TCGA database. One of these three patients did not have metastasis at the time of the study, although they had developed metastasis at the time of the latest update. The fourth patient was the only patient in the low-risk group who died from metastatic disease. Incidentally, this patient did not show either a *BAP1* or *SF3B1* mutation, nor did they have any changes in copy number for chromosome 3, all of which would be expected signs of poor prognosis. For the remaining three patients in the low risk group who developed metastasis, two showed *SF3B1* mutations, which have been associated with the development of late metastases, suggesting that methylation patterns are altered in patients who develop earlier metastases, but may be different in patients who will develop later metastases. Supporting this, 15 of the 18 patients with *SF3B1* mutations were classified in the low-risk group. As none of the patients in the low-risk group who developed metastasis showed *BAP1* mutations or copy number alterations at chromosome 3, other factors may be contributing to metastasis in these patients and warrant further examination.

For the patients in the high-risk grouping, metastasis tended to occur early (average of 1.86 years), and was generally, but not always, associated with the presence of alterations in chromosome 3 or *BAP1* mutations. As these features are associated with early metastasis, this is a potential indicator that there are alterations in methylation that may promote the development of metastasis through changes in gene expression. Additionally, as inactivating BAP1 mutations can occur across multiple regions of the gene, it is possible that not all BAP1 mutations have been detected in this study, and that changes in methylation in the high risk group may be related to changes in the BAP1 gene that were not discovered in the TCGA study.

We conducted a GO analysis to determine which genes are most commonly differentially methylated in the high-risk group. Differential methylation at a number of genes involved in cancer (KEGG pathways) and for tumor suppressors (Tables S1 and S2) may point to differential regulation of cell proliferation and tumor dissemination occurring through changes in methylation in UM in genes that are not mutated. Our analysis revealed differential methylation in multiple signaling pathways that have specifically been involved in UM progression (Figure 5), including the PI3K-Akt signaling pathway (Figure 5A, KEGG pathways, Table S4), which has been implicated in UM [30]. Particularly of interest was the hypermethylation of a number of genes involved in the negative regulation of ERK1/2 in the high-risk group (Figure 5), and the hypermethylation of 10 probes associated with the PTEN gene in the high-risk group (Table S7). PTEN has previously been demonstrated to act as a tumor suppressor in UM, and is known to have a role in the regulation of both the PI3K/Akt and ERK1/2 pathways [31]. While previous data has suggested that genomic alterations are responsible for changes in PTEN signaling in UM, methylation may also play a role in regulating PTEN expression [32]. While the MAPK pathway has been shown to be commonly activated in UM, this effect does not occur through mutations in Ras genes [33]. Instead, the constitutive activation of GNAQ/GNA11 is believed to lead to the activation of this signaling cascade [34]. Despite the lack of mutations in Ras family genes, the differential methylation at numerous genes involved in Ras signaling (Figure 5A, Table S5) indicate a potential impact of methylation on signaling in this pathway in UM. In the same vein, the *GNAQ/GNA11* mutations in UM lead to increased phosphorylation of ERK, driving growth in UM cells through the Ras/Raf/MEK/ERK pathway [35]. The hypermethylation of multiple genes involved in the negative regulation of ERK1/2 signaling in the high-risk group implies the existence of alternative mechanisms that might also be working to increase ERK activation in UM. Along with this, UM cells show upregulation of PI3K/Akt/mTOR pathway, and the methylation of genes involved in mTOR and PI3K signaling (Figure 5A, Tables S3 and S4) points to yet another example of a signaling pathway known to be altered in UM potentially being epigenetically regulated to some extent. Additionally, genes coding for one of the IL12 receptor subunits, IL12RB2, were found to be hypermethylated in the high-risk group. IL12RB2 forms a receptor with high affinity for IL12 along with IL12RB1, leading to activation of signaling. IL12RB2 is the subunit in the IL12 receptor that is required for IL12-dependent signaling [36]. Hypermethylation at these sites in the high-risk group may suggest decreased receptor activity in the high-risk cases, especially given that hypermethylation was associated with reduced gene expression in these cases (Figure 7C,F). IL-12 has shown anti-tumor activity in other cancer types. In lung cancer cell lines that are negative for IL12RB2, use of the demethylating agent 5-aza-deoxycytidine was able to restore expression of the receptor [36]. Additionally, IL12RB2 knockout mice have been shown to develop spontaneous tumors (B cell and lung epithelial), and restoring the IL12RB2 leads to reduction of these tumors (in terms of proliferation, size, and microvessel formation) [37]. Endogenous IL-12 was shown to exert antitumor effects only in IL12RB2+ tumors, suggesting that changes in gene expression associated with DNA methylation may also have an impact on host antitumor response mechanisms. Hypermethylation at genes encoding its receptor could point to a new therapeutic avenue in UM [38].

While large-scale epigenetic changes have been documented in UM tumors, and these changes have been significantly related to prognosis, there are currently no approved treatments using these agents in UM. In vitro, treatment with epigenetic modifying drugs has been shown to reduce growth and invasiveness in UM cell lines, and decitabine (a DNMT inhibitor) in combination with MEK inhibition has been shown to suppress growth in UM cells [39,40]. Additionally, decitabine has been used safely in clinical trials via hepatic arterial infusion in patients with unresectable liver metastases (NCT02316028), which is promising in the context of high risk UM patients [41].

While these agents hold promise, identification of the exact methylation events responsible for development of metastatic disease are needed to uncover clinical targets. Methylation of specific genes likely has significant impact on gene expression in UM, either through direct silencing or through affecting different pathways such as histone acetylation or ubiquitination. For example, hypermethylation of apoptosis-related genes and hypomethylation of growth promoting genes in the high-risk group may indicate potential targets for therapeutic avenues to explore. In our study, the specific DMPs associated with NFIA, HDAC4, and IL12RB2 correlated with the level of gene expression of these genes as seen on RNA sequencing data (Figure 7), with hypermethylation at these probes being associated with lower expression of transcripts for these genes [42,43]. Additionally, genes with very consistent differences in methylation between the two groups in this study such as NFIA and HDAC4 may be useful prognostic indicators for UM, and their methylation and expression patterns warrant further study. Because these genes appear to be epigenetically regulated in UM, the use of epigenetic modifying drugs could be a powerful strategy. This is consistent with the findings of Field et al. [18], showing that many specific genes and functional pathways are altered through methylation in certain UM cases, and that this information may help in the future to find potential therapeutic avenues for UM patients that have a high risk of developing metastases. As suggested, BAP1 mutations seem to be importantly associated with the altered methylation patterns, though the lack of BAP1 mutations in some patients in the high-risk group implies that there are also potentially other events that can lead to the same downstream effects on methylation patterns.

Among the many DMPs, one of the most highly differentially methylated genes was found to be NFIA. Since this gene is ubiquitously expressed in many tissues [44], this significant difference in methylation is of interest. We identified hypermethylation at CpG shores and islands along with hypomethylation at CpG shelves in the high-risk cases, suggesting that methylation may be impacting gene expression of NFIA (Figure 7A,D). The NFIA gene encodes a member of the nuclear factor 1 (NF1) family of transcription factors, and has been associated with cancer prognosis in some cancers, such as astrocytomas [45], and may be associated positively or negatively with prognosis depending on the tumor type [46]. NF1 family genes have been shown to play a role in epigenetic regulation, via remodeling of chromatin structure to alter gene expression. Depending on the tumor type, NF1 genes may act as either an oncogene or tumor suppressor, potentially through their regulatory effects on gene expression [46]. The high degree of differential methylation of this gene in the high-risk group suggests methylation of the NFIA gene as both a potential biomarker and therapeutic target in UM. In this cohort, the most highly differentially methylated NFIA probes were also fully segregated based on risk group, highlighting that methylation of this single gene may be able to classify patients into high and low-risk groups (Figure 6A). Our data show that, for these two loci, all cases in the high-risk group had methylation beta values above 0.5, and all cases in the low-risk group had methylation beta values below 0.5 (average of 0.83 vs. 0.23 for the first DMP and average of 0.83 vs. 0.31 for the second DMP).

RASSF1 was also found to be highly differentially methylated at multiple probes between the high and low-risk groups (Table S10). This gene has been investigated in UM and has been shown to be inactivated through methylation. Induction of RASSF1 expression has been shown to reduce tumorigenicity of UM cells in vitro [47]. Previously, 5-aza-2-deoxycytidine was shown to reverse RASSF1 methylation in a UM cell line, suggesting that the presence of hypermethylation in this region is a potentially reversible change that increases tumorigenicity in UM [47].

Zinc finger (ZNF) proteins are gaining interest in cancer studies due to their potential roles as either tumor suppressors or oncogenes [48]. In the present study, hypermethylation was seen in multiple ZNF genes in the high-risk group, especially ZNF358 (three probes with a log FC > 1.5, range: 3.49–4.37) and ZNF532 (nine probes with a log FC > 1.5, range: 1.63–4.04). ZNFs can function as tumor suppressors, and are inactivated in some tumor types through promoter hypermethylation [49]. ZNF358 specifically is expressed in neural folds during neural crest differentiation, and acts as a transcription factor [50]. Similarly to NFIA, multiple probes for ZNF358 segregate very strongly by risk groupings (Figure 6B). Overall, these large-scale differences in methylation of sites associated with these ZNFs suggest the potential of methylation at these sites as a biomarker of disease progression, as well as a target for epigenetic modifying drugs.

HDAC inhibitors are currently of strong clinical interest in UM due to their potential to reverse the phenotypic effects of loss of BAP1 expression, specifically inducing growth arrest and differentiation in UM [25]. Additionally, recent work by Kuznetsov et al. noted a relationship between BAP1 loss and HDAC4, where BAP1 mutant UM cells showed a change in HDAC4 expression pattern from cytoplasmic to nuclear. It was hypothesized that this change was at least partially responsible for restricting the function of HDAC4 in these cells [51]. This furthers the idea that HDAC4 specifically might be an important histone deacetylase in UM progression. In our analysis, 49 probes associated with HDAC were found to be hypomethylated in the high-risk group, including probes with a log fold change of up to 4.08 between the two risk groups (sample of differentially methylated CpGs, Figure 6C). Of note, hypomethylation of several sites associated with HDAC4 was also seen. While current studies involving use of valproic acid, an HDAC inhibitor, as adjuvant therapy in UM are underway (NCT02068586), inhibitors which selectively target HDAC4 should also be investigated to determine whether all HDAC inhibitors are equally promising options as possible adjuvant treatments in UM.

Given the relatively short patient follow up, this study was not able to show a pattern of methylation that predicts late metastasis. Further studies with longer follow up times should be performed to determine whether late metastasis shows a separate methylation pattern.

## 5. Conclusions

To conclude, patterns of DNA methylation in this cohort were significantly related to prognosis. Our analysis reveals that hierarchical clustering of methylation values separates 80 UM cases into two major groups that differ very significantly in terms of the development of metastasis and overall survival. Additionally, there are very significant differences between the two groups in methylation of specific genes known to be important in UM and in cancer progression in general, including PTEN, NFIA, IL12RB2, RASSF1, and HDAC4. These observed changes point to a role of methylation analysis, both for wide scale changes and for individual loci, as being potentially useful for prognostication of patients as well as offering insight into the potentially reversible changes that are driving UM towards a metastatic phenotype. Furthermore, the observation that specific loci were able to effectively separate the two groups in the same manner as the hierarchical clustering based on 10,000 loci suggests that changes in methylation may be observable in samples that contain very small amounts of DNA. Given the high rate of metastasis and its associated poor prognosis, such data provide important insight into novel and clinically useful biomarkers and therapeutic targets in UM.

## Figures and Tables

**Table 1:** Patient characteristics.

Number of Patients	80
Sex (M:F)	45:35
Number of Deaths	24
Age at diagnosis (average, years (range))	62 (22–86)
Follow up (average, days (range))	767 (4–2600)

**Table 2:** Patient characteristics by group.

	Low-Risk	High-Risk	
Number of Patients	40	40	
Sex (M:F)	22:18	23:17	* Χ^2^: 0.0508, *p* = 0.822
Age at diagnosis (average, years (range))	59 (22–79)	65 (41–86)	** *p* = 0.423
Follow up (average, days (range))	973 (6–2600)	560 (4–1862)	
Metastasis	4	23	* Χ^2^: 20.18, *p* < 0.00001
Death (from metastasis or unspecified)	1	23	* Χ^2^: 28.81, *p* < 0.00001

* Chi Square test: n = 80 df = 1, ** Fisher’s exact test.

**Table 3:** Tumor characteristics by group.

	Low-Risk	High-Risk	Significance
Tumor thickness (mm)	9.99	10.9	*p* = 0.171
Tumor diameter (mm)	16.15	17.72	*p* = 0.044
Cell type (% spindle:epithelioid)	~80:20	~50:50	
Presence of closed connective loops	13	30	X^2^ = 14.5, *p* = 0.00013
Extraocular extension	2	5	X^2^ = 1.4, *p* = 0.23

**Table 4:** Mutations and chromosomal aberrations by group.

Genetic Alteration	Low-Risk	High-Risk	Significance
*GNAQ* mutations	25	15	* Χ^2^: 5, *p* = 0.0253
*GNA11* mutations	14	22	* Χ^2^: 3.23, *p* = 0.0722
*BAP1* mutation	0	35	** *p* < 0.00001
Chromosome 3 loss	3	35	* Χ^2^: 51.33, *p* < 0.00001
Chromosome 6p gain	33	12	* Χ^2^: 22.4, *p* < 0.00001
Chromosome 8q gain	23	37	* Χ^2^: 13.06 *p* = 0.0003
Chromosome 1 loss	7	11	* Χ^2^: 1.147, *p* = 0.284

* Chi Square test: n = 80 df = 1, ** Fisher’s exact test.

## Supplementary Materials

The following are available online at https://www.mdpi.com/2075-1729/10/10/248/s1, Table S1: Differentially methylated genes in the Pathways in Cancer KEGG pathway with a log FC ≥ 1.5, Table S2: Differentially methylated genes in the Tumor Suppressors KEGG pathway with a log FC ≥ 1.5, Table S3: Differentially methylated genes in the mTOR signaling KEGG pathway with a log FC ≥ 1.5, Table S4: Differentially methylated genes in the PI3K-Akt KEGG pathway with a log FC ≥ 1.5, Table S5: Differentially methylated genes in the RAS signaling KEGG pathway with a log FC ≥ 1.5, Table S6: Differentially methylated genes in the Negative Regulation of ERK1/2 Signaling KEGG pathway with a log FC ≥ 1.5, Table S7: Log Fold Change for all differentially methylated probes associated with the PTEN gene, Table S8: Log Fold Change for all differentially methylated probes associated with the IL12RB2 gene, Table S9: Log Fold Change for all differentially methylated probes associated with the NFIA gene, Table S10: Log Fold Change for all differentially methylated probes associated with the RASSF1 gene, Table S11: Log Fold Change for all differentially methylated probes associated with the ZNF358 gene, Table S12: Log Fold Change for all differentially methylated probes associated with the ZNF532 gene, Table S13: Log Fold Change for all differentially methylated probes associated with the HDAC4 gene
