# Supplementary material for: Novel Methylation Patterns Predict Outcome in Uveal Melanoma
Source: Life (Basel). 2020 Oct 20;10(10):248. doi: 10.3390/life10100248 (PMC7589184; doi:10.3390/life10100248)
Supplement: Supplementary file 1 [file life-10-00248-s001.pdf]

life

MDPI

Article

Novel Methylation Patterns Predict Outcome in Uveal Melanoma

Sarah Tadhg Ferrier [1] and Julia Valdemarin Burnier [1,2,3,*]

1 Cancer Research Program, Research Institute of the McGill University Health Centre, Montreal, QC, Canada, H4A 3J1; sarah.ferrier@mail.mcgill.ca
2 Experimental Pathology Unit, Department of Pathology, McGill University; Montreal, QC, Canada, H3A 0G4
3 Department of Oncology, McGill University; Montreal, QC, Canada, H3A 0G4
* Correspondence: Julia.burnier@mcgill.ca

Table S1. Differentially methylated genes in the Pathways in Cancer KEGG pathway with a log FC ≥ 1.5.

ID	Gene Name	Species	Differentially Methylated Probes	Log Fold Change (High vs Low Risk)	Adjusted P Value	Average Beta Value, Low	Average Beta Value, High
**ABL1**	ABL proto-oncogene 1, non-receptor tyrosine kinase(ABL1)	Homo sapiens	cg13440206,	−1.85238	1.39E−06	0.576589	0.259088
			cg02915920	−1.84042	8.03E−06	0.482846	0.192714
			cg21195763	1.685721	3.13E−19	0.573548	0.83358
**ADCY2**	adenylate cyclase 2(ADCY2)	Homo sapiens	cg14116052	2.454448	4.3E−24	0.513149	0.885217
**ADCY6**	adenylate cyclase 6(ADCY6)	Homo sapiens	cg25196508	3.480923	2.9E−25	0.188362	0.792499
**AKT1**	AKT serine/threonine kinase 1(AKT1)	Homo sapiens	cg14116052	2.454448	4.3E−24	0.513149	0.885217
**BMP4**	bone morphogenetic protein 4(BMP4)	Homo sapiens	cg08046044	1.527233	3.98E−06	0.049923	0.209543
			cg01873886	1.789942	2.55E−05	0.026254	0.1723

**CDKN1B**	cyclin dependent kinase inhibitor 1B(CDKN1B)	Homo sapiens	cg06197769	3.78761	1.9E−30	0.187364	0.82662
**CTNNA3**	catenin alpha 3(CTNNA3)	Homo sapiens	cg09538287	2.037243	5.62E−09	0.251955	0.539339
**DAPK3**	death associated protein kinase 3(DAPK3)	Homo sapiens	cg22304239	1.939735	1.18E−13		
**EDNRB**	endothelin receptor type B(EDNRB)	Homo sapiens	cg18210860	2.815506	1.87E−14	0.444524	0.810551
			cg24785726	3.163564	2.54E−15	0.080629	0.539854
			cg23326536	3.195834	6.45E−17	0.071876	0.541248
			cg13866767	3.463542	1.49E−19	0.28389	0.815008
**EPAS1**	endothelial PAS domain protein 1(EPAS1)	Homo sapiens	cg21276379	1.824026	5.68E−14	0.186755	0.437118
			cg17091312	2.112437	2.76E−23		
**ETS1**	ETS proto-oncogene 1, transcription factor(ETS1)	Homo sapiens	cg25244476	2.507626	9.92E−07	0.25583	0.602367
**FGF1**	fibroblast growth factor 1(FGF1)	Homo sapiens	cg15371881	3.061708	4.38E−25	0.219393	0.69523
**FZD6**	frizzled class receptor 6(FZD6)	Homo sapiens	cg10929921	3.957203	3.22E−19	0.333365	0.912152
**GNA11**	G protein subunit alpha 11(GNA11)	Homo sapiens	cg02261780	2.268738	2.51E−16	0.353174	0.732472
			cg24366168	2.349167	6.69E−12	0.43427	0.894937
			cg00168191	2.478771	1.2E−11	0.510916	0.876102
			cg13527233	2.144414	1.19E−11	0.540858	0.863307
**GNAI1**	G protein subunit alpha i1(GNAI1)	Homo sapiens	cg05424060	1.904542	2.69E−07		
**GNB4**	G protein subunit beta 4(GNB4)	Homo sapiens	cg01750654	1.647758	4.02E−11	0.594913	0.827501
			cg03466124	1.862402	3.31E−08	0.108087	0.324521

**GNG11**	G protein subunit gamma 11(GNG11)	Homo sapiens	cg06439941	2.094159	1.67E−09	0.218242	0.565099
			cg08038054	2.335425	3.13E−11	0.069817	0.348166
			cg02914800	3.14371	1.04E−14	0.10902	0.602134
**GSTP1**	glutathione S-transferase pi 1(GSTP1)	Homo sapiens	cg22224704	2.261199	1.11E−08	0.19543	0.520111
			cg11566244	3.087685	6.52E−12	0.134838	0.555003
**IGF1R**	insulin like growth factor 1 receptor(IGF1R)	Homo sapiens	cg11852333	1.967845	3.57E−14	0.258781	0.568105
**ITGA3**	integrin subunit alpha 3(ITGA3)	Homo sapiens	cg12127162	1.833962	2.92E−16	0.254277	0.538208
**ITGAV**	integrin subunit alpha V(ITGAV)	Homo sapiens	cg03192874	2.961775	2.21E−15		
**KRAS**	KRAS proto-oncogene, GTPase(KRAS)	Homo sapiens	cg23152216	2.293392	5.96E−27	0.470784	0.837552
**LPAR6**	lysophosphatidic acid receptor 6(LPAR6)	Homo sapiens	cg03646329	2.221789	8.4E−09	0.105173	0.345011
**MAPK8**	mitogen-activated protein kinase 8(MAPK8)	Homo sapiens	cg19612574	1.694113	4.14E−15	0.113088	0.332177
**MAPK9**	mitogen-activated protein kinase 9(MAPK9)	Homo sapiens	cg09663237	2.203989	3.06E−06	0.584195	0.883176
**MMP2**	matrix metallopeptidase 2(MMP2)	Homo sapiens	cg00355286	1.518071	7.31E−10	0.183672	0.439098
**PDGFRA**	platelet derived growth factor receptor alpha(PDGFRA)	Homo sapiens	cg02273436	1.911305	1.17E−06	0.160494	0.467324
**PIK3R1**	phosphoinositide–3-kinase regulatory subunit 1(PIK3R1)	Homo sapiens	cg01251150	2.237358	1.26E−17	0.060815	0.28225

**PLEKHG5**	pleckstrin homology and RhoGEF domain containing G5(PLEKHG5)	Homo sapiens	cg07181374	1.727515	1.69E−17		
**PML**	promyelocytic leukemia(PML)	Homo sapiens	cg01947066	1.959361	1.08E−06		
**PPARD**	peroxisome proliferator activated receptor delta(PPARD)	Homo sapiens	cg26668919	2.934903	3.68E−17		
**PRKCA**	protein kinase C alpha(PRKCA)	Homo sapiens	cg14121185	−2.67592	1.62E−20	0.874693	0.429846
			cg05115424	1.608107	7.51E−10	0.081959	0.220044
			cg09655520	1.820661	1.39E−09	0.166933	0.419827
**PTEN**	phosphatase and tensin homolog(PTEN)	Homo sapiens	cg26127345	1.535502	0.001508	0.429051	0.633389
			cg19358349	1.702888	0.000271	0.401807	0.644288
			cg01228636	1.712449	0.000372	0.491384	0.734546
			cg19634213	1.721931	0.00022	0.40649	0.663402
			cg04616691	1.78722	0.000905	0.364272	0.561777
			cg03236184	1.901193	7.83E−05	0.438936	0.706127
**RARB**	retinoic acid receptor beta(RARB)	Homo sapiens	cg21902772	2.206659	9.56E−18	0.291665	0.708268
			cg23518541	3.4762	1.05E−16	0.096579	0.60235
**RASSF1**	Ras association domain family member 1(RASSF1)	Homo sapiens	cg02930432	1.590468	2.75E−09	0.197452	0.462608
			cg21418575	1.510304	9.39E−08	0.335072	0.572985
			cg07130266	2.266083	3.24E−09	0.249514	0.57952
**RB1**	RB transcriptional corepressor 1(RB1)	Homo sapiens	cg13221796	1.972518	2.27E−20		
**RXRB**	retinoid X receptor beta(RXRB)	Homo sapiens	cg07931652	1.523593	4.19E−17	0.524678	0.77896

**SLC2A1**	solute carrier family 2 member 1(SLC2A1)	Homo sapiens	cg22025263	2.001813	8.33E−11	0.510382	0.81735
**SMAD3**	SMAD family member 3(SMAD3)	Homo sapiens	cg04809597	1.97252	1.92E−23	0.05713	0.252782
**SUFU**	SUFU negative regulator of hedgehog signaling(SUFU)	Homo sapiens	cg09119776	1.824803	1.59E−16	0.068277	0.233147
**TCF7L1**	transcription factor 7 like 1(TCF7L1)	Homo sapiens	cg20693690	−1.5635	3.97E−07	0.533941	0.240248
			cg22834443	1.837207	1.9E−12	0.548775	0.843847
			cg20693690	−1.5635	3.97E−07	0.533941	0.240248
**TCF7L2**	transcription factor 7 like 2(TCF7L2)	Homo sapiens	cg07168930	1.804367	1.5E−14	0.712649	0.911392
			cg03339956	2.395134	3.93E−26	0.218402	0.600015
**TGFB2**	transforming growth factor beta 2(TGFB2)	Homo sapiens	cg11976166	−1.8673	2.93E−05	0.442588	0.167974
			cg10484211	2.167142	1.63E−13	0.08019	0.33298
			cg13285637	−1.8383	6.64E−05	0.424687	0.162007
			cg06899755	−1.54824	0.000222	0.447125	0.173171
			cg22021178	−1.50285	0.000406	0.359804	0.125219

Table S2. Differentially methylated genes in the Tumor Suppressors KEGG pathway with a log FC ≥ 1.5.

	Gene Name	Species	Differentially Methylated Probes	Log Fold Change (High vs Low Risk)	Adjusted P Value	Average Beta Value, Low	Average Beta Value, High

**ATM**	ATM serine/threonine kinase(ATM)	Homo sapiens	cg14761454	2.171709	5.33E−11	0.113337	0.410785
**CDKN1B**	cyclin dependent kinase inhibitor 1B(CDKN1B)	Homo sapiens	cg06197769	3.78761	1.9E−30	0.187364	0.82662
**CDKN1C**	cyclin dependent kinase inhibitor 1C(CDKN1C)	Homo sapiens	cg17265994	1.60087	5.24E−15	0.098808	0.272309
			cg03143849	1.697443	4.88E−16	0.380944	0.710675
**CDKN2D**	cyclin dependent kinase inhibitor 2D(CDKN2D)	Homo sapiens	cg25685983	1.766552	7.18E−23	0.110149	0.323895
**DAB2**	DAB2, clathrin adaptor protein(DAB2)	Homo sapiens	cg12290311	3.117633	5.95E−21	0.09455	0.555251
**DAPK3**	death associated protein kinase 3(DAPK3)	Homo sapiens	cg22304239	1.939735	1.18E−13		
**DIS3L2**	DIS3 like 3'–5' exoribonuclease 2(DIS3L2)	Homo sapiens	cg14802771	2.279833	3.93E−17	0.193749	0.543178
**DLC1**	DLC1 Rho GTPase activating protein(DLC1)	Homo sapiens	cg18792365	2.274905	5.82E−10	0.185786	0.565794
			cg22045977	2.575186	2.44E−15	0.407574	0.828947
**EFNA1**	ephrin A1(EFNA1)	Homo sapiens	cg07207669	1.829389	7.93E−07	0.308278	0.606053
**FES**	FES proto-oncogene, tyrosine kinase(FES)	Homo sapiens	cg25647583	1.620561	1.83E−12	0.337424	0.613609
			cg26405020	1.775185	3.08E−16	0.103383	0.313802
			cg09397246	1.7864	4.28E−11	0.038035	0.194971
**HIF3A**	hypoxia inducible factor 3 alpha subunit(HIF3A)	Homo sapiens	cg12068280	2.287241	3.28E−19	0.23857	0.656095

**HPGD**	hydroxyprostaglandin dehydrogenase 15-(NAD)(HPGD)	Homo sapiens	cg03772063	2.169526	7.27E−13	0.544367	0.843968
**MCC**	mutated in colorectal cancers(MCC)	Homo sapiens	cg02528008	2.18208	3.41E−17	0.138006	0.447979
**MTUS1**	microtubule associated tumor suppressor 1(MTUS1)	Homo sapiens	cg00121389	2.338386	4.67E−18	0.289024	0.711872
			cg01993952	3.183913	3.56E−18	0.331804	0.792952
			cg23002008	3.231493	8.86E−18	0.394688	0.876206
			cg12548824	3.485792	2.2E−25	0.250505	0.83362
			cg18563140	3.558719	8.99E−23		
**PINX1**	PIN2/TERF1 interacting, telomerase inhibitor 1(PINX1)	Homo sapiens	cg26886855	2.717973	1.02E−10		
**PML**	promyelocytic leukemia(PML)	Homo sapiens	cg01947066	1.959361	1.08E−06		
**PRKCD**	protein kinase C delta(PRKCD)	Homo sapiens	cg08170911	2.447478	8.68E−26	0.173984	0.533695
**PTEN**	phosphatase and tensin homolog(PTEN)	Homo sapiens	cg26127345	1.535502	0.001508	0.429051	0.633389
			cg19358349	1.702888	0.000271	0.401807	0.644288
			cg01228636	1.712449	0.000372	0.491384	0.734546
			cg19634213	1.721931	0.00022	0.40649	0.663402
			cg04616691	1.78722	0.000905	0.364272	0.561777
			cg03236184	1.901193	7.83E−05	0.438936	0.706127
**RASSF1**	Ras association domain family member 1(RASSF1)	Homo sapiens	cg02930432	1.590468	2.75E−09	0.197452	0.462608
			cg07130266	2.266083	3.24E−09	0.249514	0.57952
			cg21418575	1.510304	9.39E−08	0.335072	0.572985

			cg21372200	1.510504	3.36E−08	0.329236	0.588495
			cg06117233	1.592409	1.03E−08	0.240456	0.475515
			cg06980053	1.753488	7.45E−09	0.27348	0.555539
**RASSF4**	Ras association domain family member 4(RASSF4)	Homo sapiens	cg11753018	1.574196	2.82E−09	0.522055	0.784378
			cg24483689	1.967374	7.13E−17	0.475436	0.788271
**RB1**	RB transcriptional corepressor 1(RB1)	Homo sapiens	cg13221796	1.972518	2.27E−20		
			cg17419299	2.21785	3.31E−21		
			cg19473653	2.335442	1.98E−23		
**RB1CC1**	RB1 inducible coiled-coil 1(RB1CC1)	Homo sapiens	cg15973818	2.328448	5.92E−23	0.35814	0.755258
**SASH1**	SAM and SH3 domain containing 1(SASH1)	Homo sapiens	cg22685901	2.204937	4.66E−14	0.460327	0.832441
**SIK1**	salt inducible kinase 1(SIK1)	Homo sapiens	cg19135247	2.488609	7.04E−11	0.100361	0.473306
**STARD13**	StAR related lipid transfer domain containing 13(STARD13)	Homo sapiens	cg16223976	1.697226	8.07E−15	0.451039	0.743896
**STK11**	serine/threonine kinase 11(STK11)	Homo sapiens	cg16601904	2.278019	7.92E−13	0.274959	0.621749
**SUFU**	SUFU negative regulator of hedgehog signaling(SUFU)	Homo sapiens	cg09119776	1.824803	1.59E−16	0.068277	0.233147
**TSC1**	tuberous sclerosis 1(TSC1)	Homo sapiens	cg14211075	1.914518	5.87E−18	0.421238	0.780034
**WWOX**	WW domain containing oxidoreductase(WWOX)	Homo sapiens	cg01623475	−2.46666	5.04E−11	0.761141	0.327081
			cg04254167	2.54376	3.56E−12	0.193268	0.586033

**ZDHHC17**	zinc finger DHHC-type containing 17(ZDHHC17)	Homo sapiens	cg18837713	2.451172	2.91E−24	0.328923	0.801488

Table S3. Differentially methylated genes in the mTOR signaling KEGG pathway with a log FC ≥ 1.5

.

ID	Gene Name	Species	Differentially Methylated Probes	Log Fold Change (High vs Low Risk)	Adjusted P Value	Average beta Value, Low	Average Beta Value, High
**AKT1**	AKT serine/threonine kinase 1(AKT1)	Homo sapiens	cg14116052	2.454448	4.3E−24	0.513149	0.885217
**CAB39L**	calcium binding protein 39 like(CAB39L)	Homo sapiens	cg01618923	1.76264	4.39E−13	0.616829	0.876456
**IRS1**	insulin receptor substrate 1(IRS1)	Homo sapiens	cg05263838	1.69305	4.84E−07	0.230883	0.485612
**PTEN**	phosphatase and tensin homolog(PTEN)	Homo sapiens	cg26127345	1.535502	0.001508	0.429051	0.633389
			cg19358349	1.702888	0.000271	0.401807	0.644288
			cg01228636	1.712449	0.000372	0.491384	0.734546
			cg19634213	1.721931	0.00022	0.40649	0.663402
			cg04616691	1.78722	0.000905	0.364272	0.561777
			cg03236184	1.901193	7.83E−05	0.438936	0.706127
**PIK3R1**	phosphoinositide−3-kinase regulatory subunit 1(PIK3R1)	Homo sapiens	cg01251150	2.237358	1.26E−17	0.060815	0.28225
**PRKCA**	protein kinase C alpha(PRKCA)	Homo sapiens	cg14121185	−2.67592	1.62E−20	0.874693	0.429846

**STK11**	serine/threonine kinase 11(STK11)	Homo sapiens	cg16601904	2.278019	7.92E−13	0.274959	0.621749
**TSC1**	tuberous sclerosis 1(TSC1)	Homo sapiens	cg14211075	1.914518	5.87E−18	0.421238	0.780034
**ULK3**	unc−51 like kinase 3(ULK3)	Homo sapiens	cg20801110	1.87354	2.79E−14	0.131913	0.501952

Table S4. Differentially methylated genes in the PI3K-Akt signaling KEGG pathway with a log FC ≥ 1.5

**ID**	**Gene Name**	**Species**	**Differentially Methylated Probes**	**Log Fold Change (High vs Low Risk)**	**Adjusted P Value**	**Average Beta Value, Low**	**Average Beta Value, High**
**AKT1**	AKT serine/threonine kinase 1(AKT1)	Homo sapiens	cg14116052	2.454448	4.3E−24	0.513149	0.885217
**ANGPT1**	angiopoietin 1(ANGPT1)	Homo sapiens	cg18031596	1.632527	2.79E−07	0.459754	0.702836
**ATF6B**	activating transcription factor 6 beta(ATF6B)	Homo sapiens	cg11580844	1.503817	9.54E−09	0.567255	0.798651
			cg12273368	1.507859	2.01E−05	0.157889	0.347245
			cg26037142	1.73427	1.27E−07	0.093327	0.302313
**BCL2L11**	BCL2 like 11(BCL2L11)	Homo sapiens	cg06223562	2.960699	2.06E−15	0.427113	0.867355
**CCND3**	cyclin D3(CCND3)	Homo sapiens	cg12151296	1.692885	8.55E−11	0.037846	0.113328
			cg00374492	1.772585	5.58E−23	0.077127	0.287289
			cg19297231	2.509268	9.23E−32	0.041721	0.283341
**CDKN1B**	cyclin dependent kinase inhibitor 1B(CDKN1B)	Homo sapiens	cg06197769	3.78761	1.9E−30	0.187364	0.82662
**COL11A1**	collagen type XI alpha 1 chain(COL11A1)	Homo sapiens	cg03520644	1.618823	4.82E−08	0.031405	0.196742
			cg00172849	1.999957	4.41E−09	0.055192	0.274217

			cg26913669	2.102304	2.44E−13	0.598955	0.89671
**COL11A2**	collagen type XI alpha 2 chain(COL11A2)	Homo sapiens	cg08733307	2.660359	8.15E−18	0.47069	0.943783
			cg04770813	−1.64486	0.000256		
			cg16634404	1.505328	6.48E−16	0.136766	0.339358
			cg26385062	1.533797	8.69E−14	0.126167	0.302528
			cg02266086	1.534744	1.18E−15	0.050627	0.183503
			cg21893764	1.662155	6.59E−13	0.600363	0.84737
			cg22320183	1.767212	8.55E−18	0.420494	0.728267
			cg15407213	1.859479	1.05E−11	0.526713	0.812844
			cg14683730	1.902269	5.28E−16	0.601684	0.877669
			cg17560929	2.002801	9.49E−18	0.260409	0.597083
**CREB3L4**	cAMP responsive element binding protein 3 like 4(CREB3L4)	Homo sapiens	cg09895920	2.307801	3.32E−10	0.30769	0.759773
**CREB5**	cAMP responsive element binding protein 5(CREB5)	Homo sapiens	cg23281552	−2.15895	1.45E−13	0.56621	0.166395
			cg03226887	1.513661	1.5E−06	0.307877	0.535686
			cg22681186	1.76285	2.16E−09	0.191216	0.455499
**EFNA1**	ephrin A1(EFNA1)	Homo sapiens	cg07207669	1.829389	7.93E−07	0.308278	0.606053
**FGF1**	fibroblast growth factor 1(FGF1)	Homo sapiens	cg15371881	3.061708	4.38E−25	0.219393	0.69523
			cg17593472	1.862401	4.58E−19	0.103875	0.337684
			cg19954000	2.476565	5.38E−24	0.110559	0.444379
**FOXO3**	forkhead box O3(FOXO3)	Homo sapiens	cg04855107	2.656668	6.13E−16	0.422016	0.833628
			cg21184115	2.092368	6.11E−20	0.629905	0.886342
			cg08792630	2.964245	3.28E−14		

**G6PC3**	glucose−6-phosphatase catalytic subunit 3(G6PC3)	Homo sapiens	cg26534477	1.86973	2.34E−11	0.31365	0.618814
			cg18426551	1.894664	8.07E−10	0.154885	0.405578
			cg22243039	2.547672	4.45E−15	0.179281	0.560702
**GNB4**	G protein subunit beta 4(GNB4)	Homo sapiens	cg01750654	1.647758	4.02E−11	0.594913	0.827501
			cg03466124	1.862402	3.31E−08	0.108087	0.324521
**GNG11**	G protein subunit gamma 11(GNG11)	Homo sapiens	cg06439941	2.094159	1.67E−09	0.218242	0.565099
			cg02914800	3.14371	1.04E−14	0.10902	0.602134
			cg08038054	2.335425	3.13E−11	0.069817	0.348166
**IGF1R**	insulin like growth factor 1 receptor(IGF1R)	Homo sapiens	cg11852333	1.967845	3.57E−14	0.258781	0.568105
**IRS1**	insulin receptor substrate 1(IRS1)	Homo sapiens	cg05263838	1.69305	4.84E−07	0.230883	0.485612
**ITGA3**	integrin subunit alpha 3(ITGA3)	Homo sapiens	cg12127162	1.833962	2.92E−16	0.254277	0.538208
**ITGAV**	integrin subunit alpha V(ITGAV)	Homo sapiens	cg03192874	2.961775	2.21E−15		
**ITGB4**	integrin subunit beta 4(ITGB4)	Homo sapiens	cg07436562	2.221927	1.07E−25	0.274514	0.657516
**KRAS**	KRAS proto-oncogene, GTPase(KRAS)	Homo sapiens	cg23152216	2.293392	5.96E−27	0.470784	0.837552
**LPAR6**	lysophosphatidic acid receptor 6(LPAR6)	Homo sapiens	cg03646329	2.221789	8.4E−09	0.105173	0.345011
**PDGFRA**	platelet derived growth factor receptor alpha(PDGFRA)	Homo sapiens	cg02273436	1.911305	1.17E−06	0.160494	0.467324
**PIK3R1**	phosphoinositide−3-kinase regulatory subunit 1(PIK3R1)	Homo sapiens	cg01251150	2.237358	1.26E−17	0.060815	0.28225
**PKN1**	protein kinase N1(PKN1)	Homo sapiens	cg03626511	1.795673	9.46E−12	0.412789	0.70717
**PPP2R3A**	protein phosphatase 2 regulatory subunit B''alpha(PPP2R3A)	Homo sapiens	cg23194766	3.347877	2.96E−27	0.155295	0.722266
**PRKCA**	protein kinase C alpha(PRKCA)	Homo sapiens	cg14121185	−2.67592	1.62E−20	0.874693	0.429846

**PTEN**	phosphatase and tensin homolog(PTEN)	Homo sapiens	cg26127345	1.535502	0.001508	0.429051	0.633389
			cg19358349	1.702888	0.000271	0.401807	0.644288
			cg01228636	1.712449	0.000372	0.491384	0.734546
			cg19634213	1.721931	0.00022	0.40649	0.663402
			cg04616691	1.78722	0.000905	0.364272	0.561777
			cg03236184	1.901193	7.83E−05	0.438936	0.706127
**SPP1**	secreted phosphoprotein 1(SPP1)	Homo sapiens	cg15460348	2.747597	1.08E−11	0.418663	0.838071
			cg20261167	2.937721	1.39E−12	0.460194	0.885425
**STK11**	serine/threonine kinase 11(STK11)	Homo sapiens	cg16601904	2.278019	7.92E−13	0.274959	0.621749
**THEM4**	thioesterase superfamily member 4(THEM4)	Homo sapiens	cg09063149	1.639568	4.13E−10		
			cg26347189	1.92722	5.09E−12		
**TNXB**	tenascin XB(TNXB)	Homo sapiens	cg09114581	−1.77386	1.03E−06	0.331392	0.102144
			cg13823701	−1.72872	1.04E−10	0.783831	0.471462
			cg19268434	−1.70504	8.14E−06	0.466698	0.212354
			cg18417373	−1.59536	6.52E−09	0.212665	0.063564
			cg15540749	−1.53238	3.03E−09		
			cg21337909	1.516293	1.25E−06	0.420422	0.668582
			cg01337207	1.525985	2.84E−06	0.091661	0.219728
			cg10365886	1.813212	1.56E−06	0.081412	0.199427
**TSC1**	tuberous sclerosis 1(TSC1)	Homo sapiens	cg14211075	1.914518	5.87E−18	0.421238	0.780034
**YWHAQ**	tyrosine 3-monooxygenase/tryptophan 5-monooxygenase activation protein theta(YWHAQ)	Homo sapiens	cg15748006	1.764721	2.69E−10	0.120031	0.339017

Table 5. Differentially methylated genes in the RAS signaling KEGG pathway with a log FC ≥ 1.5.

ID	Gene Name	Species	Differentially Methylated Probes	Log Fold Change (High vs Low Risk)	Adjusted P Value	Average beta Value, Low	Average Beta Value, High
**ABL1**	ABL proto-oncogene 1, non-receptor tyrosine kinase(ABL1)	Homo sapiens	cg13440206,	−1.85238	1.39E−06	0.576589	0.259088
			cg02915920	−1.84042	8.03E−06	0.482846	0.192714
**AKT1**	AKT serine/threonine kinase 1(AKT1)	Homo sapiens	cg14116052	2.454448	4.3E−24	0.513149	0.885217
**ANGPT1**	angiopoietin 1(ANGPT1)	Homo sapiens	cg03245734	1.885287	3.19E−07	0.300807	0.572671
			cg18031596	1.632527	2.79E−07	0.459754	0.702836
**EFNA1**	ephrin A1(EFNA1)	Homo sapiens	cg07207669	1.829389	7.93E−07	0.308278	0.606053
**ETS1**	ETS proto-oncogene 1, transcription factor(ETS1)	Homo sapiens	cg25244476	2.507626	9.92E−07	0.25583	0.602367
			cg03295554	1.542659	2.23E−09	0.40522	0.685169
**ETS2**	ETS proto-oncogene 2, transcription factor(ETS2)	Homo sapiens					
**EXOC2**	exocyst complex component 2(EXOC2)	Homo sapiens	cg14219317	−2.4566	8.53E−13	0.791947	0.35102
			cg26889659	−2.06602	4.46E−08	0.942684	0.736261
			cg09607658	2.586518	5.1E−21		
**FGF1**	fibroblast growth factor 1(FGF1)	Homo sapiens	cg15371881	3.061708	4.38E−25	0.219393	0.69523

**GNB4**	G protein subunit beta 4(GNB4)	Homo sapiens	cg01750654	1.647758	4.02E−11	0.594913	0.827501
			cg03466124	1.862402	3.31E−08	0.108087	0.324521
**GNG11**	G protein subunit gamma 11(GNG11)	Homo sapiens	cg06439941	2.094159	1.67E−09	0.218242	0.565099
			cg02914800	3.14371	1.04E−14	0.10902	0.602134
			cg08038054	2.335425	3.13E−11	0.069817	0.348166
**IGF1R**	insulin like growth factor 1 receptor(IGF1R)	Homo sapiens	cg11852333	1.967845	3.57E−14	0.258781	0.568105
**KRAS**	KRAS proto-oncogene, GTPase(KRAS)	Homo sapiens	cg23152216	2.293392	5.96E−27	0.470784	0.837552
**MAPK8**	mitogen-activated protein kinase 8(MAPK8)	Homo sapiens	cg19612574	1.694113	4.14E−15	0.113088	0.332177
**MAPK9**	mitogen-activated protein kinase 9(MAPK9)	Homo sapiens	cg09663237	2.203989	3.06E−06	0.584195	0.883176
**PAK1**	p21 (RAC1) activated kinase 1(PAK1)	Homo sapiens	cg14076258	2.244198	9.31E−17	0.249832	0.669792
**PDGFRA**	platelet derived growth factor receptor alpha(PDGFRA)	Homo sapiens	cg02273436	1.911305	1.17E−06	0.160494	0.467324
**PIK3R1**	phosphoinositide−3-kinase regulatory subunit 1(PIK3R1)	Homo sapiens	cg01251150	2.237358	1.26E−17	0.060815	0.28225
**PLA1A**	phospholipase A1 member A(PLA1A)	Homo sapiens	cg06068392	3.589907	6.18E−20	0.282764	0.853068
**PRKCA**	protein kinase C alpha(PRKCA)	Homo sapiens	cg14121185	−2.67592	1.62E−20	0.874693	0.429846

			cg05115424	1.608107	7.51E−10	0.081959	0.220044
			cg09655520	1.820661	1.39E−09	0.166933	0.419827
**RAB5C**	RAB5C, member RAS oncogene family(RAB5C)	Homo sapiens	cg00435173	1.705061	8.56E−12	0.055333	0.200904
**RAPGEF5**	Rap guanine nucleotide exchange factor 5(RAPGEF5)	Homo sapiens	cg12894814	2.365007	7.45E−13	0.578418	0.915151
**RASSF1**	Ras association domain family member 1(RASSF1)	Homo sapiens	cg02930432	1.590468	2.75E−09	0.197452	0.462608
			cg07130266	2.266083	3.24E−09	0.249514	0.57952
			cg21418575	1.510304	9.39E−08	0.335072	0.572985
			cg21372200	1.510504	3.36E−08	0.329236	0.588495
			cg06117233	1.592409	1.03E−08	0.240456	0.475515
			cg06980053	1.753488	7.45E−09	0.27348	0.555539
**SHC4**	SHC adaptor protein 4(SHC4)	Homo sapiens	cg26337669	1.812095	7.98E−19	0.527409	0.896985
			cg26814712	2.746152	9.7E−19	0.085799	0.427092

Table 6. Differentially methylated genes in the Negative Regulation of ERK1/2 signaling KEGG pathway with a log FC ≥ 1.5.

ID	Gene Name	Species	Differentially Methylated Probes	Log Fold Change (High vs Low Risk)	Adjusted P Value	Average beta Value, Low	Average Beta Value, High
**CNKSR3**	CNKSR family member 3(CNKSR3)	Homo sapiens	cg08927844	−2.27644	2.97E−14	0.876441	0.497476
			cg16175941	2.252762	4.73E−16	0.501376	0.853069
**CSK**	c-src tyrosine kinase(CSK)	Homo sapiens	cg13578134	1.70682	1.71E−10	0.060061	0.34615

**DLG1**	discs large MAGUK scaffold protein 1(DLG1)	Homo sapiens	cg17696750	1.827729	1.99E−10	0.500693	0.829287
**GSTP 1**	glutathione S-transferase pi 1(GSTP1)	Homo sapiens	cg22224704	2.261199	1.11E−08	0.19543	0.520111
			cg09038676	2.666525	9.53E−13	0.029387	0.289375
**ITGB1 BP1**	integrin subunit beta 1 binding protein 1(ITGB1BP1)	Homo sapiens	cg09734418	−1.51915	0.000166	0.808651	0.578962
			cg09761705	1.733371	2.04E−06	0.693193	0.930472
**LYN**	LYN proto-oncogene, Src family tyrosine kinase(LYN)	Homo sapiens	cg06818377	1.645783	1.02E−12	0.157167	0.390638
			cg11982546	2.602661	4.44E−21	0.122589	0.522136
**PTEN**	phosphatase and tensin homolog(PTEN)	Homo sapiens	cg26127345	1.535502	0.001508	0.429051	0.633389
			cg19358349	1.702888	0.000271	0.401807	0.644288
			cg01228636	1.712449	0.000372	0.491384	0.734546
			cg19634213	1.721931	0.00022	0.40649	0.663402
			cg04616691	1.78722	0.000905	0.364272	0.561777
			cg03236184	1.901193	7.83E−05	0.438936	0.706127
**PTPR R**	protein tyrosine phosphatase, receptor type R(PTPRR)	Homo sapiens	cg01432552	1.861903	4.44E−15	0.495372	0.805973
**SPRY 1**	sprouty RTK signaling antagonist 1(SPRY1)	Homo sapiens	cg15206981	2.101061	3.68E−08	0.21633	0.553251
			cg08251025	2.421152	9.1E−10	0.227702	0.613871
			cg21353144	2.543733	1.01E−09	0.338308	0.71798
			cg26449178	1.634087	4.19E−06	0.687759	0.883531

**SPRY 2**	sprouty RTK signaling antagonist 2(SPRY2)	Homo sapiens	cg22369786	1.795045	1.47E−08	0.080262	0.293171
			cg15374435	1.880112	8.11E−08	0.182656	0.447754
**TNIP1**	TNFAIP3 interacting protein 1(TNIP1)	Homo sapiens	cg12642815	2.204535	6.58E−17		
			cg22480558	2.571383	7.84E−23		
**WNK2**	WNK lysine deficient protein kinase 2(WNK2)	Homo sapiens	cg20616414	−2.46363	2.2E−08	0.292862	0.037877
			cg10272601	−1.86566	2.66E−05	0.170371	0.013493
			cg21194517	1.849301	3.85E−11		

Table S7. Log Fold Change for all differentially methylated probes associated with the PTEN gene

Name	Relation_to_Island	UCSC_RefGene_Name	LogFC
**cg03236184**	Island	PTEN;KILLIN	1.901193
**cg04616691**	Island	PTEN;KILLIN	1.78722
**cg19634213**	Island	PTEN;KILLIN	1.721931
**cg01228636**	Island	PTEN;KILLIN	1.712449
**cg19358349**	Island	PTEN;KILLIN	1.702888
**cg26127345**	Island	PTEN;KILLIN	1.535502
**cg04824711**	N_Shore	PTEN;KILLIN	1.444914
**cg25452974**	Island	PTEN;KILLIN	1.436399
**cg08995089**	Island	PTEN;KILLIN	1.393284
**cg27084903**	Island	PTEN;KILLIN	1.357835

Table S8. Log Fold Change for all differentially methylated probes associated with the IL12RB2 gene

Name	Relation_to_Island	UCSC_RefGene_Name	LogFC
**cg00318756**	N_Shore	IL12RB2	4.074567
**cg19745415**	N_Shore	IL12RB2	4.035874
**cg06952660**	N_Shore	IL12RB2	3.993203
**cg01356829**	N_Shore	IL12RB2	3.974715
**cg11132246**	N_Shore	IL12RB2	3.111831
**cg12633410**	OpenSea	IL12RB2	2.705041
**cg03975876**	N_Shore	IL12RB2;IL12RB2	2.656201
**cg00993824**	N_Shore	IL12RB2;IL12RB2	1.361834
**cg27416067**	N_Shore	IL12RB2;IL12RB2	1.31938
**cg02566391**	OpenSea	IL12RB2	−1.15142

Table S9. Log Fold Change for all differentially methylated probes associated with the NFIA gene

Name	Relation_to_Island	UCSC_RefGene_Name	LogFC
**cg18946602**	S_Shore	NFIA;NFIA;NFIA;NFIA	4.173637
**cg16001865**	OpenSea	NFIA;NFIA;NFIA;NFIA	3.492806
**cg12262372**	N_Shore	NFIA	3.188973
**cg12126901**	OpenSea	NFIA;NFIA;NFIA;NFIA	2.355875
**cg19097500**	N_Shore	NFIA	1.588947
**cg10628205**	N_Shore	NFIA;NFIA;NFIA;NFIA	1.487113
**cg19899972**	OpenSea	NFIA;NFIA;NFIA;NFIA	1.128364
**cg15546227**	N_Shelf	NFIA;NFIA	−1.58506
**cg14093103**	N_Shelf	NFIA;NFIA	−2.20013

Table S10. Log Fold Change for all differentially methylated probes associated with the RASSF1 gene

Name	Relation_to_Island	UCSC_RefGene_Name	LogFC
**cg07130266**	Island	RASSF1;RASSF1;RASSF1;RASSF1;RASSF1	2.266083
**cg06980053**	S_Shore	RASSF1;RASSF1;RASSF1;RASSF1	1.753488

**cg06117233**	Island	RASSF1;RASSF1;RASSF1;RASSF1;RASSF1	1.592409
**cg02930432**	Island	RASSF1;RASSF1;RASSF1;RASSF1	1.590468
**cg21372200**	S_Shore	RASSF1;RASSF1;RASSF1;RASSF1	1.510504
**cg21418575**	Island	RASSF1;RASSF1;RASSF1;RASSF1	1.510304
**cg06821120**	Island	RASSF1;RASSF1;RASSF1;RASSF1	1.470988
**cg09386807**	Island	RASSF1;RASSF1;RASSF1;RASSF1	1.354297
**cg20826201**	Island	RASSF1;RASSF1;RASSF1;RASSF1	1.283362
**cg25486143**	Island	RASSF1;RASSF1	1.008904

Table S11. Log Fold Change for all differentially methylated probes associated with the ZNF358 gene.

Name	Relation_to_Island	UCSC_RefGene_Name	LogFC
**cg00351537**	N_Shore	ZNF358	4.366471
**cg02026535**	N_Shore	ZNF358	4.199227
**cg19921377**	N_Shore	ZNF358	3.489339

Table S12. Log Fold Change for all differentially methylated probes associated with the ZNF532 gene.

Name	Relation_to_Island	UCSC_RefGene_Name	LogFC
**cg06487082**	N_Shore	ZNF532;ZNF532	4.044402
**cg12406559**	N_Shore	ZNF532;ZNF532	3.646373
**cg12737497**	Island	ZNF532	3.240236
**cg03096126**	Island	ZNF532	2.879877
**cg26172016**	N_Shore	ZNF532	2.635631
**cg24243629**	S_Shore	ZNF532	2.479404
**cg04212150**	Island	ZNF532	2.378976
**cg24049621**	N_Shore	ZNF532	1.967654
**cg14269096**	S_Shore	ZNF532	1.636641

Table S13. Log Fold Change for all differentially methylated probes associated with the HDAC4 gene.

Name	Relation_to_Island	UCSC_RefGene_Name	cFC
**cg19955928**	N_Shore	HDAC4	1.046703
**cg26876664**	OpenSea	HDAC4	−1.02396
**cg23971806**	OpenSea	HDAC4	−1.03939

**cg13781718**	N_Shore	HDAC4	−1.03996
**cg19449565**	OpenSea	HDAC4	−1.07381
**cg21996813**	N_Shelf	HDAC4	−1.07961
**cg21827163**	OpenSea	HDAC4	−1.11055
**cg21963406**	OpenSea	HDAC4	−1.12288
**cg07618780**	OpenSea	HDAC4	−1.13964
**cg06915343**	N_Shore	HDAC4	−1.14881
**cg02381790**	OpenSea	HDAC4	−1.16423
**cg25103905**	OpenSea	HDAC4	−1.19481
**cg03886681**	N_Shore	HDAC4	−1.20492
**cg14758976**	OpenSea	HDAC4;MGC16025	−1.20535
**cg03475776**	S_Shore	HDAC4	−1.20543
**cg20924425**	OpenSea	HDAC4	−1.22822
**cg01771737**	S_Shelf	HDAC4	−1.27317
**cg00630775**	N_Shore	HDAC4	−1.27928
**cg12595293**	OpenSea	HDAC4	−1.29593
**cg27074582**	S_Shore	HDAC4	−1.30743
**cg05903736**	S_Shore	HDAC4	−1.31141
**cg07609703**	N_Shore	HDAC4	−1.3183
**cg21656251**	N_Shore	HDAC4	−1.417
**cg19125791**	OpenSea	HDAC4	−1.46509
**cg23870168**	OpenSea	HDAC4	−1.49104
**cg11534215**	S_Shelf	HDAC4	−1.54146
**cg01942751**	N_Shore	HDAC4	−1.54933
**cg11122767**	OpenSea	HDAC4	−1.56615
**cg08177681**	OpenSea	HDAC4	−1.58758
**cg06533788**	OpenSea	HDAC4	−1.61493
**cg01647308**	OpenSea	HDAC4	−1.62883
**cg27144223**	S_Shelf	HDAC4	−1.68685
**cg20149840**	OpenSea	HDAC4	−1.699
**cg23949574**	OpenSea	HDAC4	−1.73597

**cg16360836**	OpenSea	HDAC4	−1.8192
**cg05114739**	OpenSea	HDAC4	−1.82017
**cg18479711**	N_Shore	HDAC4	−1.83972
**cg19246018**	N_Shore	HDAC4	−1.84819
**cg27587095**	S_Shore	HDAC4	−1.86322
**cg11707035**	OpenSea	HDAC4	−2.03859
**cg18736431**	OpenSea	HDAC4	−2.12299
**cg25828346**	S_Shore	HDAC4	−2.15728
**cg26641275**	N_Shelf	HDAC4	−2.60834
**cg20706597**	S_Shore	HDAC4	−2.65649
**cg10515232**	S_Shore	HDAC4	−2.67711
**cg21190228**	OpenSea	HDAC4	−2.92946
**cg26627327**	OpenSea	HDAC4	−3.04717
**cg17410431**	OpenSea	HDAC4	−3.21471
**cg10071550**	OpenSea	HDAC4	−4.08402
